# 65 &THRIVE: TRANSFORMING OLDER ADULT CARE IN THE EMERGENCY DEPARTMENT

**DOI:** 10.1093/geroni/igac059.2750

**Published:** 2022-12-20

**Authors:** Tracey Vien, Stella Bobroff

**Affiliations:** Kaiser Permanente Med Group/Southern California, Los Angeles, California, United States; Kaiser Permanente, Los Angeles, California, United States

## Abstract

Kaiser Permanente Los Angeles Medical Center’s (LAMC) Utilization Management Department expanded our geriatric initiative throughout the hospital with emphasis in the Emergency Department (ED). The ED is the first point of patient contact in the hospital, early identification and initiation of interventions needed for potential discharge barriers will assist in timely discharges. LAMC obtained Geriatric ED Accreditation (GEDA) Level 2 this year, the first in the Los Angeles County to achieve this title. This was done by implementing ten new policies with quality improvement (QI) metrics, conducting age sensitivity trainings for our staff, and enhancing our ED rooms to be geriatric friendly. Two of the ten interventions in our ED include 1) volunteers to provide support to our geriatric patients prevent functional decline, prevent delirium and readmission rates, and 2) palliative care consults initiated in the ED prevent suffering and promote the best possible quality of life for patients who are facing a serious illness. Between 2020 and 2021, there has been a 15% increase for the census of 65+ patients. 21% increase of number of older adults with ED readmissions, and a 13% increase in number of older adults staying in the ED for more than 8 hours. With the increasing older adult population, we see a rise in our admission and readmissions of older adults in our ED. This indicates a need for geriatric specialized care and Kaiser plans to obtain GEDA for all Southern California Hospitals.

